# Medical Suggestions for the Treatment of Dysentery, of Intermittent and Remittent Fevers, as Generally Prevalent at Certain Seasons among Troops in the Field

**Published:** 1817-03

**Authors:** 


*Medical Suggestions for the Treatment of Dysentery, of intermittent and remittent Fevers, as generally prevalent at certain Seasons among Troops in the Field.* By E. S. Somers, M. D. &c. Physician in Chief to the Allied Armies in the Peninsula. 8vo. pp. 78. London, 1816.

The subject of this slender volume is one of no trifling interest to the leaders of fleets and armies, as well as to medical practitioners. Many a well-planned expedition has failed, in consequence of the ravages of dysentery alone; and more of our brave countrymen were swept off by this scourge, during the peninsular war, than by the sword of the enemy. Several plans of treatment have been recommended by different authors of late years; all the result of experience, and each insisted on as the best and surest means of checking this Herculean malady. But those who have studied, with most care, the pathological nature of dysentery, are well convinced, that we may arrive at the same point by very different routes; and that there is no one plan of treatment applicable to all cases and to all climates. The *principal* mean of cure in one case will become the *auxiliary* in another; and he only will be able to combine the different remedial measures, or direct any one of them singly with advantage, who has a correct knowledge and enlightened view of the *ratio symp-*

*tomatum* and *post mortem* appearances in this severe affliction.

Our author had charge of the NUNCIO General Hospital at Lisbon during two years, where three hundred of the worst cases of dysentery and intermittent fevers were usually collected. But moderate success attended the usual modes of treatment in the *former* disease, and chronic states of the complaint or visceral affections were the too common consequences. These discouraging results led Dr. Somers to reflect, that

"—blood taken suddenly from the arm was likely to prove less oppressive, and exhausting to the system, than a continual oozing of an undefined quantity from a considerable portion of extravasating surface."

On this basis was founded a practice of blood-letting in dysentery, unprecedented for its freedom in the annals of this distemper. In every recent case, therefore, recourse was had to immediate venæsection, which was repeated, at certain intervals, as long as blood continued to be voided with the stools, due regard being paid to the state of the pulse.

"Success almost universal, and beyond the most sanguine expectation, was the grateful result of the adventurous practice." p. 3.

Dr. M'Gregor then recommended the practice throughout the whole medical department of the army—

"—and wheresoever employed, it was almost as uniformly rewarded with decided success." ib.

We do not know how Dr. Somers can reconcile this last statement with Dr. M'Gregor's document in the sixth volume of the Medico-Chirurgical Transactions, where it is asserted, that the peninsular army lost, in less than three years, 4717 men by dysentery alone! (Vide *Med. Ch. Journal*, vol. ii. page 142.) Yet we do not urge this as any objection to the depleting practice in dysentery, knowing, as we do, its utility; but only to shew, that no *methodus medendi* has yet been able to curb the disease, in the manner represented in the above passages by Dr. Somers.

*Intermittent fever.* Dr. Somers says, that he purposely avoids the *fashionable* discussion of the *proximate cause* of febrile paroxysms. We could have excused the no less fashionable habit of making long quotations from authors, of passages which every Tyro can repeat by rote. Who would believe, that a *practical* writer would open his lean octavo of 70 or 80 pages, with *eleven pages* verbatim from the text of Cullen! The predisponent and occasional causes offer nothing new. To cut short the cold fit, or put it off altogether, let the patient, says the Doctor, take a table spoonful of the mistura emetica every quarter of an hour, until full vomiting be produced, drinking, during the operation, "large repeated draughts of *mawkishly* warm camomile infusion." 15. After this the warm bath, and a warm bed, with diluent drinks, will bring on the sweating stage, and finally terminate the paroxysm. As soon as the circulation becomes calm, and the heat of the body reduced to its natural standard, the following draught should be given every two hours: Infus. cinchon. ℥j, pulv. cinchon. ℈iv, tinct. cinchon. ʒij. M. Horse exercise, generous diet, and a few glasses of old Port wine at dinner, as adjuvants. Whenever the patient feels a presentiment of an approaching cold fit, he is to take the "Haustus Prophylacticus"—Liq. ammon. acet. ℥jß, tinct. opii ♏. L, sp. æth. sulph. ʒj, sp. lavend. ʒß. M. and repair to bed. This course of treatment is to be persevered in till two or three periods of the apprehended attack shall have gone by, when the haustus tonicus may be gradually lowered, and ultimately left off.

In chronic intermittent, Dr. S. advises a course of mercury, as visceral disease generally accompanies the complaint. He never observed any decided or permanent effect from arsenic. In this, our own experience does not lead us to coincide with our author; for we have seen the most unequivocal cures performed by this mineral.

*Remittent fever.* From the numerous cases in which Dr. S. has witnessed "the marvellous good effects" of the cold water affusion, in bringing on an immediate remission, he cannot help considering this remedy "as of *sovereign power* and efficacy in the cure of remittent fever." The remission being *thus* procured, the *haustus tonicus* is to be administered as in the intermittent form. With respect to evacuations, "emollient and gently laxative injections" are all that our author recommends! We need not at this day point out the inadequacy of such practice in *remittent fever.* The general voice of the profession, and our own experience, have long ago convinced us, that Dr. Somers's plan is any thing but judicious; any thing but effectual in that most direful scourge of soldiers and sailors, in marshy situations and autumnal seasons. Need we refer again to the melancholy experience gained on the shores of the Scheldt? Could not any of Dr. Somers's brother officers have pointed out to him the inefficacy of his plan on such trying occasions? Has Dr. Somers not deigned to peruse the writings of any modern author on the disease in question?

Dysentery. We shall not transpose the symptomatology of dysentery from the pages of Dr. Somers to those of our Journal. It may be found in Cullen's *Nosology*, The Physician's *Vade-Mecum*, or any other "Compendium of Medical Practice," Mr. Bedingfield's included. The occasional cause is considered by our author to be "cold applied to the surface of the body," in which we agree with him; but not so in the following piece of antiquated, perhaps erroneous pathology:

"By such means, sensible and insensible perspiration are [is] suppressed and thrown inwards upon the alimentary canal, where, from the existing peculiar state of the *atmosphere*, it acquires a certain degree of *acrimony*, becomes *virus sui generis*, exciting local *inflammation* in the superficial blood-vessels, and *consequent* hæmorrhage." 32.

We believe that few physiologists or pathologists of the present day consider dysentery as a mere *metastasis* of perspiration from the skin to the intestines; but rather that it is a change of balance in the circulation, and that the *increased action* in the vessels of the bowels is vicarious of the *torpor* in those of the surface. As for the formation of a *virus sui generis* in the intestines, from the peculiar state of the *atmosphere*, it is worse, a thousand times, than the *nova pestis* from Bulam; and we were not prepared to find such obsolete *theories* vamped up anew by a *practical* gentleman, who sets out, in *Italics* too, with a sneer at the "fashionable discussion of *proximate causes*!" Alas! how hard a task is it to "know thyself!" But the pathology is not merely antiquated; it is erroneous. We assert, that, granting the existence of local inflammation, it does not follow, as a matter of course, that there should be — *consequent* hæmorrhage. Of all the effects, concomitants, or consequences of inflammation, *hæmorrhage* is the rarest. How often do we meet with hæmorrhage in phrenitis, pleuritis, pulmonitis, gastritis, hepatitis, or enteritis? Let Dr. Somers answer this question.

The *Methodus Medendi* of our author, however, is much more interesting and satisfactory.

"Sixteen ounces of blood were immediately drawn from the arm: in about an hour afterwards, the patient was directed to take, at three short intervals, solutio laxativa, (sulph. magnes. fruct. tamarind. et aquæ hordeat) and during the operation plentiful draughts of warm apple, rice, or barley water, and to clothe in flannel shirt, drawers, and woollen stockings. At night, a general warm bath for fifteen or twenty minutes was administered." P. 34.

Ventilation and cleanliness were very properly enjoined, and the instant removal of excrements; but why Dr. Somers should think it necessary that these excrements should be "buried *at a distance, some depth under ground,*" when he expressly denies the disease to be contagious, is an enigma which we leave our readers to solve. The fumigation too of the wards, "by means of Dr. Smyth's nitric gas," is surely a remnant of that contagious creed which he justly stigmatizes. There is inconsistency in this. Next day the venæsection is renewed; and shortly after, fifteen grains of the pulv. ipecac. c. which dose is repeated every hour for three periods. The patient meanwhile is placed naked between the blankets, under additional covering, and directed to encourage profuse sweating for ten or twelve hours, and to dilute with abundance of warm barley decoction, linseed tea, or rice water. The third morning, if blood continued to be discharged, venæsection was repeated, with the laxative solution; and at night three grains of calomel, one of opium, and one of antimonial powder. Fourth day, if the stools are still bloody, eight or ten ounces are to be taken from the arm, the pulv. ipecac. c. to be repeated. After this period venæsection was seldom necessary, though Dr. S. thinks that while blood appears in the stools the lancet should not be sheathed. The warm bath invariably relieved the excruciating pain in the bowels, and the distressing tenesmus. Starch glysters were also found serviceable; and in extreme cases, flannel swathes to the abdomen, moistened with warm camphorated spirit, or tincture of opium. In combinations of recent dysentery with intermittent fever, the latter was disregarded till the former was subdued by the means above detailed. The tone, health, and strength, were gradually restored by riding exercise, nourishing diet, and a moderate allowance of generous wine.

Many cases of dysentery, as may be expected, arrive at hospital stations, "in so advanced a stage of *chronicism*" (we suppose the others are in a state of *acuteism*) that the period for venæsection is gone by. Yet, even here, where the pulse is good, and the stools are tinged with blood, Dr. S. recommends small bleedings every second or third day, giving the calomel and opium bolus every night for three nights, and the laxative solution on the fourth morning.

It may not be out of place here to remark, that many cases of long standing chronic dysentery, which were apparently improving, the pulse, respiration, and excretions being regularly carried on, "most unexpectedly sunk, in the course of an hour, into dissolution." P. 40. What would be expected after such events in a public military hospital? Anatomical investigation without doubt. But no! The gentleman who ridicules the fashionable discussion of proximate causes, instead of a *pathological fact* presents us with a *medico-military* hypothesis!

"In the endeavour to account for the suddenness of those deaths, it might, perhaps, be no unfair inference, that the fatal *stroke* has so rapidly closed the scene, from the immediate contact of acrimonious matter having gradually *sapped* the nobler viscera, the important *mounds* of the constitution; some of these suddenly *breaking away*, an overpowering extravasation of fluid ensues, and forthwith *sweeps off* the miserable victim." P. 40.

We believe, that since the dialogues between Corporal Trim and Uncle Toby, there never was so grand a *military* elucidation of a *philosophical* subject, offered to the learned world, as Dr. Somers has here favoured us with. Let us figure to our *imaginations* that important mound of the constitution," the *stomach*, "gradually *sapped*" by that wicked pioneer, the brandy bottle, and "suddenly *breaking* away," while "an overpowering extravasation of fluid" "sweeps off" the neighbouring viscera of the abdomen, and lays the citadel of life in ruins!

But this is too melancholy a subject on which to exercise our wit; and it were inexcusable, did not the vague terms and loose analogies of our author deserve some raillery that may deter others from falling into the same improper path. In no science are precision of language and clearness of ideas more necessary than in ours; and if so, such extravaganzas as the foregoing, are highly censurable in the mouth of an old army physician, dictating to the juniors of his corps.

Dr. Somers observes, that he has seen dysenteric patients imbibe the contagion of typhus; "but never a single case of any fever that had caught the dysentery by contagion." This is conclusive evidence. In many cases of fatal chronic dysentery that Dr. S. had seen opened, the appearances on dissection were a never-failing comment and document on the bad consequences that had ensued from the omission of free blood-letting in the recent stages. The coats of the intestinal tube were found greatly injured, more especially those of the colon and rectum.

"The liver, in most cases, shewed traces of foregone inflammation, with considerable enlargement; and the spleen was generally increased in size." P. 47.

Dr. S. here introduces an interesting letter from our respected friend, Inspector of hospitals, Hennen, who gives a very favourable testimony to the efficacy of blood-letting in dysentery.

"Venæsection," says this able and experienced officer, "continued as long as severe pain on pressing the abdomen remained, or as long as florid blood appeared in the stools, I have found, without any manner of question, to be the most powerful remedy I ever used in the disease. Its effects are, to lower the fever, produce an universal perspiration, and relieve the local inflammation, which our dissections have proved to exist in *all* cases of incipient dysentery. P. 48.—"The liver is found, in every state and stage of inflammation, from a blush on the peritoneal coat up to gangrene; but in the spleen, the purulent stage is not so soon induced; its spongy substance favours an accumulation of blood, hence enlargements of it are more frequent than suppuration." P. 52.

We are of opinion, that the peninsular experience in dysentery has established the point of *copious blood-letting* being a powerful auxiliary, if not the principal mean of cure, especially among that class of society on whom the experiment was tried; but that "full and early venæsection, followed up by the plan of treatment here detailed, is the *desideratum, the specific itself* for the cure of dysentery," we deny, from very ample experience. Different constitutions and climates require variety of treatment; and where the disease, as is too often the case, is connected with organic and functional disease of the glandular viscera of the abdomen, *mercury* must take the precedence of venæsection. Both these measures will, in fact, be often necessary in the same case, and the preponderance must be left to the judgement of the practitioner. That Dr. Somers's plan is not entirely his own invention, might be proved by numerous writers from Sydenham downwards. Our coadjutor, Dr. Johnson, expressly states in his work, that, "when blood appears alarmingly in the stools, whether the fever run high or not, venæsection may be employed, without the smallest apprehension of that bug-bear, *debility*."—*On*

*Hot Climates*, p. 371.—Nevertheless, we think the profession indebted to the author of the work under review, for having proved the utility and the safety of what may be termed *extreme bleeding*, in a disease which was too generally placed out of the pale of the lancet. Dr. S. has thus contributed his mite towards the demolition of that immense fabric of error and thraldom, founded on the sandy basis of *debility* and putrescency; but which now dissolves before the light of reason and experience, like mists and darkness before the beams of a powerful sun.

